# Establishing a Gross Primary Productivity Model by SIF and PRI on the Rice Canopy

**DOI:** 10.34133/plantphenomics.0144

**Published:** 2024-02-01

**Authors:** Zhanhao Zhang, Jianmao Guo, Shihui Han, Shuyuan Jin, Lei Zhang

**Affiliations:** ^1^ School of Ecology and Applied Meteorology, Nanjing University of Information Science & Technology, Nanjing 210044, China.; ^2^ Jiangsu Key Laboratory of Agricultural Meteorology, Nanjing 210044, China.; ^3^ National Meteorological Centre, China Meteorological Administration, Beijing 100081, China.

## Abstract

Solar-induced chlorophyll fluorescence (SIF) has shown remarkable results in estimating vegetation carbon cycles, and combining it with the photochemical reflectance index (PRI) has great potential for estimating gross primary productivity (GPP). However, few studies have used SIF combined with PRI to estimate crop canopy GPP. Large temporal and spatial variability between SIF, PRI, and GPP has also been found in remote sensing observations, and the observed PRI and SIF are influenced by the ratio of different observed information (e.g., background, direct sunlit, and shaded leaves) and the physiological state of the vegetation. In this study, the PRI and SIF from a multi-angle spectrometer and the GPP from an eddy covariance system were used to assess the ability of the PRI to enhance the SIF-GPP estimation model. A semi-empirical kernel-driven Bidirectional Reflectance Distribution Function (BRDF) model was used to describe the hotspot PRI/SIF (PRI_hs_/SIF_hs_), and a modified two-leaf model was used to calculate the total canopy PRI/SIF (PRI_tot_/SIF_tot_). We compared the accuracies of PRI_hs_/SIF_hs_ and PRI_tot_/SIF_tot_ in estimating GPP. The results indicated that the PRI_tot_+SIF_tot_-GPP model performed the best, with a correlation coefficient (*R*^2^) of the validation dataset of 0.88, a root mean square error (RMSE) of 3.74, and relative prediction deviation (RPD) of 2.71. The leaf area index (LAI) had a linear effect on the PRI/SIF estimation of GPP, but the temperature and vapor pressure differences had nonlinear effects. Compared with hotspot PRI_hs_/SIF_hs_, PRI_tot_/SIF_tot_ exhibited better consistency with GPP across different time series. Our research demonstrates that PRI is effective in enhancing SIF and PRI for estimating GPP on the rice canopy and also suggests that the two-leaf model would contribute to the vegetation index tracking the real-time crop productivity.

## Introduction

Gross primary productivity (GPP) has been used to monitor the photosynthetic and carbon sequestration capacity of terrestrial vegetation [1–3]. Accurate estimation of GPP is important for capturing vegetation growth status, understanding the distribution of global carbon sinks, and understanding the impact of terrestrial vegetation on climate change [2–5]. Solar-induced chlorophyll fluorescence (SIF), based on remote sensing technology [6], provides a tool for monitoring vegetation photosynthesis and creates new opportunities for calculating GPP [2,6–8].

However, the efficiency of sunlight energy use during photosynthesis is complex, particularly for crops. Butler [9] divided the absorbed photosynthetically active radiation into 3 pathways: photochemical quenching (Kp), fluorescence (Kf), and nonradiative dissipation or nonphotochemical quenching (NPQ) (Kn) [5]. Therefore, it is insufficient to create a simple relationship between SIF and GPP, which reflects fluorescence information and photosynthesis, to monitor GPP. In addition to fluorescence and photosynthesis, the third pathway (NPQ) absorbs sunlight energy simultaneously, and NPQ typically influences the distribution of energy between Kp and Kf. Research has shown that the photochemical reflectance index (PRI) is consistent with NPQ [8,10–13]; therefore, we can estimate GPP using PRI in combination with SIF [14].

In remote sensing, the observed canopy SIF and PRI are influenced by vegetation type, observation geometry (sun–sensor–target angle), canopy structure, and the observed ratio of shaded (which can only absorb scattered radiation) to sunlit (which can absorb both scattered and direct radiation) leaves [15], causing the relationship between SIF/PRI and GPP to vary with ecosystem type, timescale, and sky conditions [16]. Multi-angle observation is an efficient method for resolving unstable observational data caused by diffuse light and describing the anisotropy of vegetation surface reflectance caused by sunlit and shaded parts of the canopy [17,18]. This method can effectively separate the influence of the illumination environment from the physiological signals on the vegetation canopy surface. The semi-empirical kernel-driven Bidirectional Reflectance Distribution Function (BRDF) model presents the reflectance distribution at different angles as a linear superposition of the BRDF shape and canopy structure relative to the sun’s position [19]. Many studies have used the BRDF model to extract hotspot reflectance from the vegetation canopy [20–22]. However, the BRDF model assumes that the reflection of the vegetation canopy background is constant because the actual observed reflectance of noncanopy information from numerous observed angles cannot be constant [20,23]. Hall [24,25] deduced from vegetation light energy use theory that the angular variation of canopy multi-angle reflectance observed every half hour may depend on the change in the shadow fraction recorded by the sensor. Zhang et al. [26] developed a two-leaf model based on the above for distinguishing the PRI between sunlit and shaded leaves in southern China subtropical forests and found that this PRI can successfully enhance the assessment of light use efficiency capabilities. Currently, few studies have investigated the possibility of applying the BRDF and two-leaf models to SIF. Furthermore, modeling SIF in combination with the PRI to estimate GPP has not been further investigated.

In this study, we used a multi-angle spectrometer and an eddy covariance (EC) system to investigate the accuracy of the PRI-boosting SIF-GPP estimation model in a subtropical rice field in China. We used a semi-empirical kernel-driven BRDF model and a two-leaf model to describe hotspot PRI and SIF (PRI_hs_/SIF_hs_) and total canopy PRI and SIF (PRI_tot_/SIF_tot_) to build the hotspot and canopy total PRI+SIF-GPP models, respectively, and used the validation dataset to evaluate these estimation models.

## Materials and Methods

### Research area and period

Experiments were carried out at the National Climate Observatory at Shou County Meteorological Administration, Anhui Province (32°26’N, 116°47’E), which has a 20 hm^2^ experimental plot. Shou County is located in China’s north–south climate transition zone, and the average temperature was 25 °C during the observation experiment [27]. Winter wheat and medium-sized rice were used as rotation crops.

### The hyperspectral observation and vegetation index calculation

**Fig. 1.**
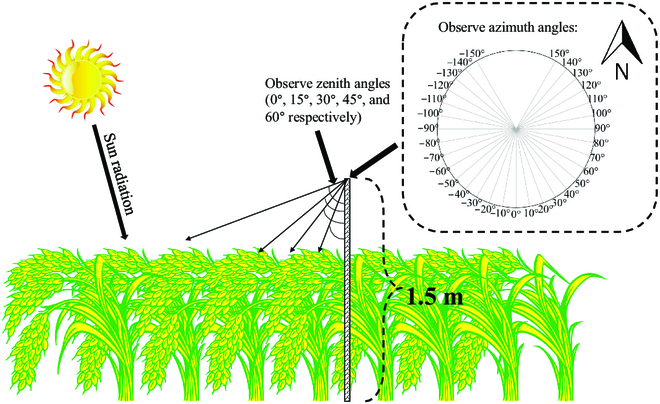
Mutil-angle hyperspectral solar and reflected irradiance measured by upper and lower spectrometer sensor at the height of 1.5 m, respectively; the lower spectrometer sensor has a fixed rotation angle both horizontally and vertically.

In the multi-angle hyperspectral observation system, a JAZ-Combo2 optical fiber spectrometer (Ocean Optics, USA), positioned 1.5 m above the experimental plot, was used to automatically observe and record irradiance, with a spectral range of 350 to 800 nm. The spectrometer had 2 fibers: one (ZFQ-15029) equipped with a cosine corrector (CC-3-UV-S) and mounted vertically upward to observe the solar irradiance at all times and the other (QP400-2-UV-VIS) connected to a Pan-Tilt Unit (PTU) platform (FLIR Systems, Goleta, CA, USA) to obtain the canopy reflected irradiance at different observation angles. Both fibers were subjected to dark-noise removal, electronic dark-noise correction, and stray-light correction by a halogen light source (HL-2000-CAL), and their average integration time is 63 ms. The bottom fiber could rotate at a speed of 10°/10 s to measure irradiance horizontally from −150° to +150° and vertically from 0° to 60° (Fig. 1). We recorded the observed zenith, azimuth, solar zenith, and solar azimuth angles as the sensor was rotated.

The hyperspectral data obtained by the JAZ-Combo2 spectrometer contained both canopy-reflected and solar irradiance. After acquiring the hyperspectral data, we used whiteboard correction to retrieve the reflectance, which is a standard diffuse radiation approach, and was calculated as follows [28,29]:

$$R=\frac{L\cdot L^{\prime }}{E\cdot E^{\prime }}$$ (1)

where $L^{\prime }$ and $E^{\prime }$ represent the absolute reflected irradiance on a standard whiteboard and solar irradiance at the same moment, respectively. *L* and *E* represent the canopy reflected irradiance and solar irradiance, respectively, measured by the spectrometer at the same moment, and *R* is the canopy reflectance.

The PRI was calculated using the reflectance retrieved by the whiteboard correction [30,31]:

$$\mathrm{PRI}=\frac{R_{531}-R_{570}}{R_{531}+R_{570}}$$ (2)

where *R*_531_ and *R*_570_ represent the reflectance at 531 nm and 570 nm, respectively.

We used the 3-band Fraunhofer Line Discrimination (3FLD) algorithm, proposed by Maier et al. [32], which uses the original dark line central band and dark line outer band to calculate the SIF. The 3FLD has a central dark line band and 2 outer dark line bands on either side of the central dark line band instead of the outer dark line band. The FLD equation is as follows:

$$\frac{E_{\text{out}}L_{\text{in}}-E_{\text{in}}L_{\text{out}}}{E_{\text{out}}-E_{\text{in}}}$$ (3)

where *L* and *E* represent the reflected canopy and solar irradiance measured by the spectrometer, respectively. The subscript “in” indicates the band at the center of the Fraunhofer dark line and “out” indicates the band outside the dark line. 3FLD uses the weighted average spectral values of the 2 outer dark line bands on either side of the center, and the equation is as follows:

Eout=ωleft·Eleft+ωright·ErightLout=ωleft·Lleft+ωright·Lright (4)

where the “left” and “right” subscripts represent the selected left and right outer bands of the dark line central band, respectively. The ω represents the weight value, which can be calculated by the wavelength (λ) of the dark line central and outer band, calculated as:

ωleft=λright−λinλright−λleftωright=λin−λleftλright−λleft (5)

The 3FLD extraction algorithm is derived by substituting Eq. 4 and 5 into the FLD algorithm (Eq. 3).

$$\frac{\left(\omega_{\text{left}}∙E_{\text{left}}+\omega_{\text{right}}∙E_{\text{right}}\right)L_{\text{in}}-E_{\text{in}}\left(\omega_{\text{left}}∙L_{\text{left}}+\omega_{\text{right}}∙L_{\text{right}}\right)}{\left(\omega_{\text{left}}∙E_{\text{left}}+\omega_{\text{right}}∙E_{\text{right}}\right)-E_{\text{in}}}$$ (6)

**Fig. 2.**
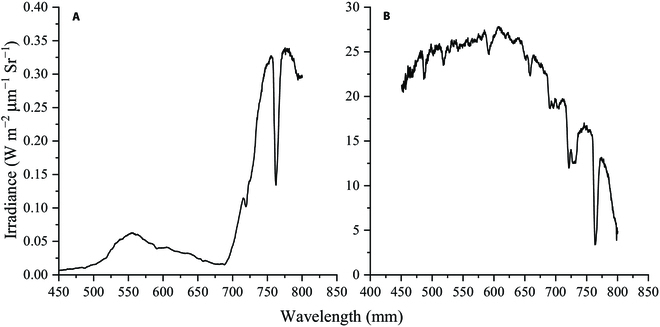
Rice canopy reflected (A) and solar (B) irradiance (W m^−2^ μm^−1^ Sr^−1^) measured by a spectrometer.

Figure 2 shows the spectra of solar irradiance and rice canopy-reflected irradiance measured by the spectrometer, which reflects the validity of the spectral data collected in this experiment. The results show that there is a decrease in the solar incident spectrum received by the rice canopy near 760 nm, and this phenomenon is most likely due to the absorption of the solar spectrum by the oxygen molecules in the atmosphere due to the presence of the O_2_-A Fraunhofer dark lines near the wavelength of 760 nm, when there is a strong absorption of oxygen in the atmosphere. Therefore, we set 762 nm as the dark line central band, and 758 and 769 nm as the corresponding left and right outer band of the dark line central band (Fig. 2).

### EC observation and GPP calculation

The EC observation system consists of a turbulence observation subsystem, a gradient observation subsystem, and a routine meteorological element technique (RMET) system. A turbulence observation subsystem was installed near the multi-angle hyperspectral observation system to measure water vapor content, CO_2_ concentration, wind speed, and temperature of the canopy. This subsystem contained an infrared open-circuit CO_2_/H_2_O gas analyzer (LI-7500, LI-Cor Inc., USA), a data collector, and a 3-dimensional ultrasonic anemometer (CSAT, Campbell Scientific, USA), which were saved and analyzed using a high-speed data collector and communication system (DATALOGGER, CR4000, Campbell Scientific, USA), with a sampling frequency of 10 Hz and taking the average of the data every 30 min. The gradient observation subsystem was installed approximately 20 m from the turbulence observation subsystem to reduce mutual interference with the EC observations, and the observation frequency was set to be synchronized with the turbulence observation subsystem. Temperature, humidity, and CO_2_ concentration were measured within and above the canopy at 5, 15, 30, 50, 80, and 200 cm above ground level using an LI-8150 (LI-Cor Inc., USA).

The raw EC data were processed using Longgernet 2.0 and Eddypro for coordinate rotation, frequency response correction, wild point rejection, and angular revision. In this study, latent heat and CO_2_ fluxes were corrected using the method of Webb et al. [33]. The average diurnal variation was used to interpolate daily data that were blank after elimination, and the Lloyd [34] respiratory equation was used to interpolate the missing night data. We used the following 4 standards to remove invalid flux data: (a) out of range of the device, (b) CO_2_ flux is negative at night, (c) 1 h before and after rainfall events, and (d) frictional wind speed below the thresholds at night (set 0.13 m·s^−1^ in our study [35]). Finally, CO_2_ flux, vapor pressure difference (VPD), and temperature (*T*) were collected every 30-min.

The CO_2_ flux measured by the EC was considered the net ecosystem exchange (NEE), which was used to calculate the GPP as follows:

$$\mathrm{GPP}=R_{e}-\mathrm{NEE}$$ (7)

where *R*_e_ is the ecological respiration rate of the experimental plots. The night NEE can be considered as *R*_e_ because there is no photosynthetically active radiation (PAR) at night and was combined with *T* to match Vant’s Hoff breath equation in this study [36,37]:

$$R_{e}=R_{e,\mathrm{ref}}\cdot e^{B\left(T-T_{\mathrm{ref}}\right)}$$ (8)

where *R*_e,ref_ is the respiration rate at the reference temperature *T*_ref_, selected as 25 °C. *R*_e,ref_ and *B* are derived by fitting Eq. 6 with NEE and *T* every half hour during non-rainy nights to NEE and *T* every half hour. The fitting results are as follows: *R*_e,ref_ = 0.22 mg·m^2^·s^−1^, *B* = 0.1235 [27].

The RMET system, supplied by VECTOR and VAISALA, was mounted approximately 5 m from the turbulence observation subsystem to measure wind direction, wind speed, total radiation (*R*_0_), and PAR above the canopy. The leaf area index (LAI) was measured by randomly sampling 10 rice plants in the field every 7 days during the observation period.

### Models

**Fig. 3.**
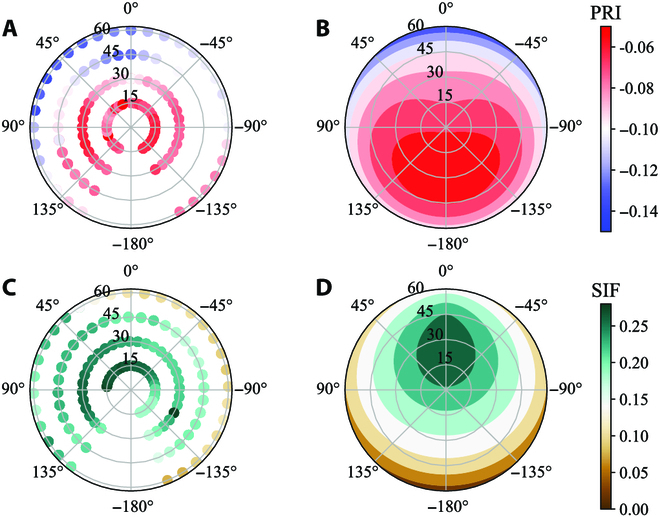
PRI (A and B) and SIF (C and D) features distribution of multi-angle (A and C) and the BRDF model (B and D).

In this study, we used a semi-empirical kernel-driven BRDF model to characterize the measured BRDF angular distributions of multi-angle PRI/SIF on the rice canopy. The solar incidence positions at different moments were calculated to extract the PRI and SIF at hotspot (PRI_hs_ and SIF_hs_) [19,38]. We modified the semi-empirical kernel-driven BRDF model first applied to the coniferous forest PRI by Hilker et al. [20], which is given as follows:

$$\begin{array}{c} \rho \left(\theta_{v},\theta_{s},∆\emptyset \right)=k_{i}+k_{g}K_{L}\left(\;\theta_{v},\theta_{s},∆\emptyset ,\frac{h}{b},\frac{b}{r}\;\right)+k_{v}K_{R}\left(\theta_{v},\theta_{s},∆\emptyset \right) \end{array}$$ (9)

where ρ refers to PRI and SIF under multi-angle; *k*_i_, *k*_g_, and *k*_v_ are different scattering components (isotropic, geometric, and volumetric); *K*_L_ and *K*_R_ are the Li-Sparse [39] and Ross–Thick [40] kernels (LSRT), which have been shown to have better application on vegetated ecosystems [41,42]. Figure 3 shows the angular distribution of multi-angle observations and BRDF modeling for PRI and SIF, and the results show that the PRI/SIF feature distribution of multi-angle is close to that of the BRDF model.

The two-leaf model proposed by Zhang et al. [26] is constructed based on a 4-scale geometric optical model [43] combined with angular normalization corrections. This model could distinguish the canopy PRI/SIF between sunlit and shaded leaves, and the total canopy PRI and SIF (PRI_tot_ and SIF_tot_) were calculated as the sum of sunlit and shaded PRI/SIF weighted by their respective sunlit and shaded LAI. The main processes of the model are as follows. First, the 4-scale model is applied to partition the canopy reflectance into 4 components at each complete multi-angle observation:

$$R_{\mathrm{can}}=R_{T}\times P_{T}+R_{G}\times P_{G}+R_{S}\times P_{S}+R_{Z}\times P_{Z}$$ (10)

where *P*_T_ and *P*_S_ represent the proportions of sunlit and shaded leaves in the canopy, respectively, and *P*_G_ and *P*_Z_ represent the corresponding proportions of sunlit and shaded leaves in the background. *R*_T_, *R*_G_, *R*_S_, and *R*_Z_ represent the reflectances of each component. Assuming that the total background proportion *P*_VG_ is the sum of *P*_G_ and *P*_Z_ at an observation angle of [43], the calculation is as follows:

$$P_{\mathrm{VG}}=e^{-0.5\Omega \times \mathrm{LAI}/\text{cos}\theta }$$ (11)

where Ω is the leaf clumping index, taken as 0.9 [44], and θ is the observed zenith angle. The sunlit leaf ratio *P*_T_ can be expressed as the ratio of canopy reflectance to leaf reflectance at a certain wavelength λ, which was 670 nm [26]. In this study, we used the PROSPECT model [45] to define leaf reflectance, and canopy reflectance was considered as the reflectance observed at each angle by multi-angle observation. Therefore, the shaded leaf ratio *P*_S_ can be calculated as follows:

$$P_{S}=1-P_{T}-P_{\mathrm{VG}}$$ (12)

because of the short duration of the angular rotation period, assuming that the PRI and SIF are constant over a period and dividing them into sunlit and shaded parts [46], as in the following equation:

$$Ρ=P_{T}\times \rho_{\mathrm{sun}}+P_{S}\times \rho_{\mathrm{sh}}$$ (13)

We used the method of Chen et al. [47] to calculate the sunlit (*L*_sun_) and shaded parts (*L*_sh_) of the LAI as follows:

$$L_{\mathrm{sun}}=2\text{cos}\theta \times \left(1-e^{\frac{-0.5\Omega \times \mathrm{LAI}}{\text{cos}\theta }}\right)$$ (14)

$$L_{\mathrm{sh}}=\mathrm{LAI}-L_{\mathrm{sun}}$$ (15)

The sunlit and shaded PRI/SIF are obtained by combining Eq. 11 using least-squares regression, and PRI_tot_/SIF_tot_ is calculated for a given moment in a rotation cycle using the weights of the *L*_sun_ and *L*_sh_:

$$\rho_{\mathrm{tot}}=\frac{L_{\mathrm{sun}}}{\mathrm{LAI}}\times \rho_{\mathrm{sun}}+\frac{L_{\mathrm{sh}}}{\mathrm{LAI}}\times \rho_{\mathrm{sh}}$$ (16)

### Data classification and model evaluation

The observations and data were collected from 2018 August 3 to October 4, and a total of 325 group datasets (25 days) were collected every half hour from 09:00 to 15:00. Each dataset contained half-hour flux data and 1,500 multi-angle canopy remote-sensing data points. By sorting the data by date and time, a modeling dataset (286 groups, 22 days) and a validation dataset (39 groups, 3 days) were created. The day of year (DOY) of the validation set was 224, 251, and 271, and these 3 dates represent the tassel, filling, and maturity stages of rice, respectively.

Multiple regression was used to perform parameter inversion based on the multi-angle PRI and SIF data collected every half hour from 09:00 to 15:00. Root-mean-square errors (RMSE) and coefficients of determination (*R*^2^) were used to evaluate the accuracy of the GPP estimation model. The RMSE was calculated as follows:

$$\text{RMSE}=\sqrt{\sum_{I=1}^{n}{\left(X_{0}-X_{m}\right)}^{2}/n}$$ (17)

where *X*_0_ is the observed value, *X*_m_ is the predicted value, and *n* is the sample size. The relative prediction deviation (RPD) [48] is used to evaluate the model’s accuracy. RPD < 1.5 suggests that the model did not have predictive power, whereas RPD > 2 suggests that the model had excellent predictive power. The RPD is calculated as follows:

$$\mathrm{RPD}=\frac{\mathrm{SD}}{\text{RMSE}}$$ (18)

where SD is the validation dataset’s standard deviation.

## Results

Vegetation indexes based on multi-angle hyperspectral observations were compared with flux data observed by the EC observation system at half-hourly and daily scales. A linear model was used to investigate the correlation between PRI/SIF and GPP at different timescales and establish GPP estimation models. The response of PRI/SIF to the environment was explored, and *R*^2^, RMSE, and RPD were calculated for the validation dataset to evaluate the accuracy of the different GPP estimation models.

### PRI, SIF, and GPP for different time series

**Fig. 4.**
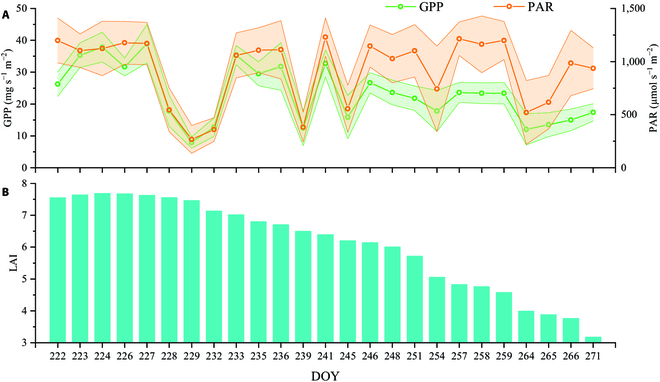
Time series of daily average GPP (mg s^−1^ m^−2^), PAR (μmol s^−1^ m^−2^) (A), and LAI (B); the error bars in (A) were the daily standard error.

**Fig. 5.**
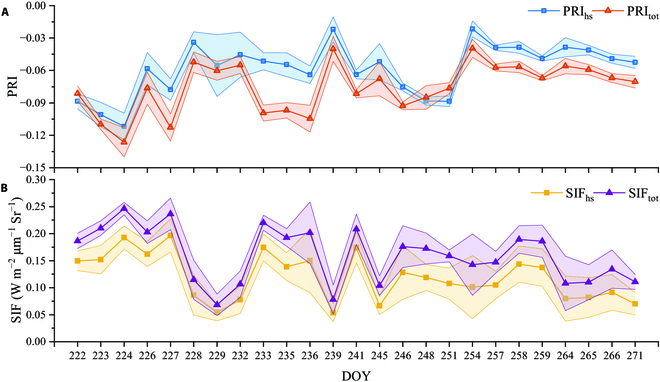
Time series of daily mean PRI_hs_ and PRI_tot_ (A), and SIF_hs_ (W m^−2^ μm^−1^ Sr^−1^) and SIF_tot_ (W m^−2^ μm^−1^ Sr^−1^) (B).

**Fig. 6.**
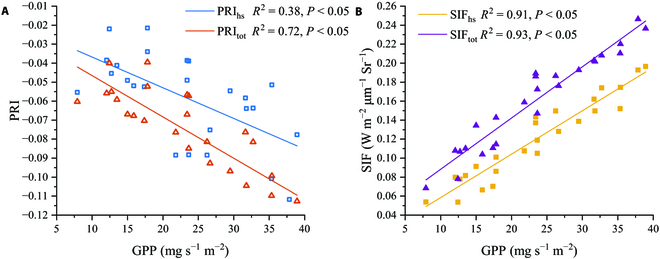
The scatterplots between daily GPP (mg s^−1^ m^−2^) and SIF_hs_/SIF_tot_ (W m^−2^ μm^−1^ Sr^−1^) (A) and PRI_hs_/PRI_tot_ (B). The lines are linear regression between for variables, and *R*^2^ is also provided.

Figure 4 shows the daily time series of the GPP, LAI, and PAR. The LAI first presented an increasing trend, reaching its peak at 7.7 at DOY 224, followed by a decrease. There was no correlation between GPP and LAI on cloudy days (daily average PAR < 1,000), whereas there was a strong correlation on clear days (daily average PAR > 1,000). Figure 5 compares the average daily SIF_tot_, SIF_hs_, PRI_tot_, and PRI_hs_ values. SIF_tot_ was greater than SIF_hs_, whereas PRI_tot_ was less than PRI_hs_. SIF effectively tracked daily changes in GPP (Fig. 5), whereas PRI could only track changes on clear days. The associations of daily mean SIFc, SIFh, PRIc, and PRIh with GPP are depicted in Fig. 6. Compared with the BRDF standardized hotspot treatment, the distinction between canopy shade and sunlit leaves in the two-leaf model improved the correlation between SIF/PRI and GPP, with the improvement in PRI being greater. PRI_tot_ and SIF_tot_ explained 72.0% and 92.8%, respectively, of the daily GPP variation.

**Fig. 7.**
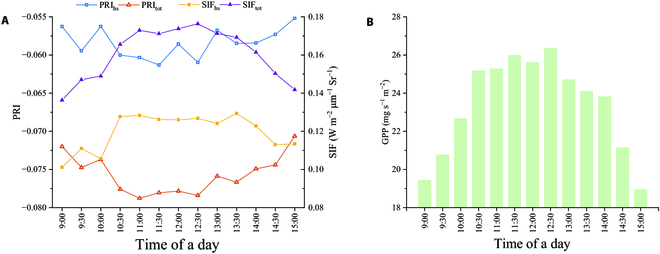
Time series of half-hourly (9:00 to 15:00 local time) SIF_hs_ (W m^−2^ μm^−1^ Sr^−1^), SIF_tot_ (W m^−2^ μm^−1^ Sr^−1^), PRI_hs_, PRI_tot_ (A), and GPP (mg s^−1^ m^−2^) (B).

**Fig. 8.**
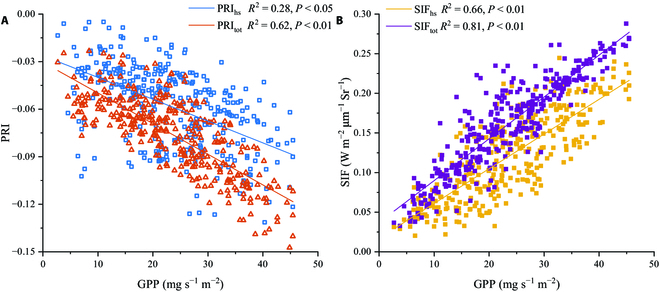
The scatterplots between half-hourly GPP (mg s^−1^ m^−2^) and SIF_hs_/SIF_tot_ (W m^−2^ μm^−1^ Sr^−1^) (A) and PRI_hs_/PRI_tot_ (B). The lines are linear regression between for variables, and the coefficient of determination (*R*^2^) is also provided.

Figure 7 compares the GPP for each half-hourly time series during the observation period using SIF_tot_, SIF_hs_, PRI_tot_, and PRI_hs_. The GPP peaked at 11:30 and 12:30, with a valley at 12:00. The SIF trended similarly to that of GPP. In contrast, the half-hourly trend of the PRI was the opposite, with 2 valleys at 11:30 and 12:30. In comparison to PRI_hs_ and SIF_hs_, the half-hourly variations in PRI_tot_ and SIF_tot_ are more parabolic, which is more consistent with the GPP trend. Figure 8 shows the correlation between GPP and the PRI/SIF half-hourly series. After accounting for the distinction between canopy shade and sun leaves, PRI_tot_ and SIF_tot_ were greater than PRI_hs_ and SIF_hs_ for estimating GPP and could explain 61.5% and 81.0% of the variation in GPP, respectively.

### Effect of different environmental stresses on PRI and SIF

**Fig. 9.**
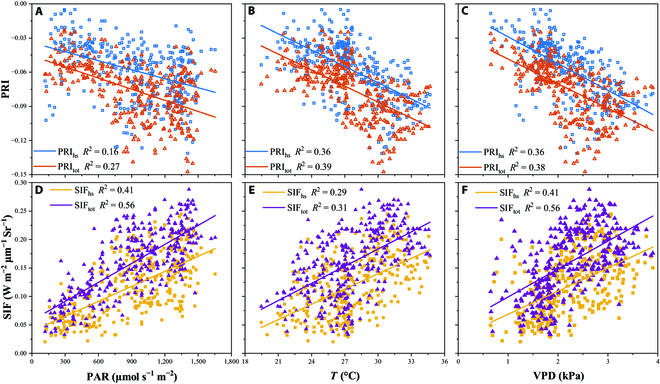
The relationship between half-hourly (9:00 to 15:00) PRI, SIF(W m^−2^ μm^−1^ Sr^−1^), and PAR(μmol s^-1^ m^-2^) (A and D), *T* (°C) (B and E), and VPD (kPa) (C and F); *R*^2^ is provided.

To explore the impact of environmental factors on the performance of the PRI and SIF estimation, the relationship between the half-hourly PRI/SIF and PAR, *T*, and VPD is shown in Fig. 9. Generally, PAR and VPD have a greater influence on SIF than PRI; *T* has a smaller impact on SIF than on PAR, but has a slightly greater impact on PRI than on PAR. Table 1 shows the correlation coefficients between GPP and PRI/SIF for different LAI, *T*, and VPD intervals. *R*^2^ was highest for LAI > 7 (*R*^2^ = 0.92), 25 °C < *T* < 30 °C (*R*^2^ = 0.79), and VPD > 3 kPa (*R*^2^ = 0.78) in terms of the relationship between SIF_tot_ and GPP. LAI had a linear effect on the PRI/SIF estimation of GPP, whereas *T* and VPD had nonlinear effects.

**Table 1.** *R*^2^ between GPP and PRI/SIF for different LAI, *T*, and VPD intervals

Factor	Interval	PRI_hs_	PRI_tot_	SIF_hs_	SIF_tot_
LAI	LAI < 6	0	0.02	0.42**	0.65**
6 < LAI < 7	0.19*	0.53**	0.58**	0.73**
LAI > 7	0.27*	0.72**	0.78**	0.92**
*T* (°C)	*T* < 25	0.04	0.06	0.46**	0.73**
25 < *T* < 30	0.07	0.53**	0.62**	0.79**
*T* > 30	0.05	0.48**	0.37**	0.59**
VPD (kPa)	VPD < 2	0	0.14*	0.49**	0.71**
2 < VPD < 3	0.15*	0.57**	0.55**	0.78**
VPD > 3	0	0.37*	0.30*	0.38**

*P < 0.05, **P < 0.01.

### Modeling and validation of PRI and SIF estimating GPP

**Fig. 10.**
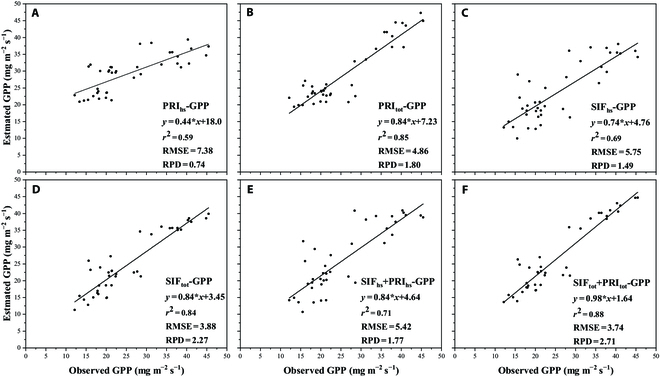
Distribution of observed and estimated values of rice GPP (mg s^−1^ m^−2^) by different estimation models (PRI_hs_-GPP (A), PRI_tot_-GPP (B), SIF_hs_-GPP (C), SIF_tot_-GPP (D), PRI_hs_+SIF_hs_-GPP (E), and PRI_tot_+SIF_tot_-GPP(F)); *R*^2^, RMSE, and RPD are provided.

As shown in Table 2, the PRI-GPP, SIF-GPP, and PRI+GPP linear estimation models were created using the respective modeling datasets. Combining PRI and SIF to estimate GPP was more accurate than using PRI and SIF individually. After the two-leaf model distinguished between shaded and sunlit leaves for the canopy, the coefficient of determination of the estimate models increased dramatically, with the PRI_tot_+SIF_tot_-GPP model having *R*^2^ = 0.86 and RMSE = 3.7. Figure 10 confirms the estimation model and uses the 6 estimation models from Table 1 to obtain GPP estimates, which form a scatter distribution with GPP observations from the validation dataset. The combination of PRI and SIF achieved better results than the estimation models of individual vegetation index (Fig. 10E and F). After distinguishing between sunlit and shaded leaves, the distribution of the observed and estimated values was closer to the 1:1 line, and the RPD was much greater than that of the PRI_hs_/SIF_hs_ estimation model. The PRI_tot_+SIF_tot_-GPP model performed the best with *R*^2^ = 0.88, RMSE = 3.8, and RPD = 2.71. The validation results showed that the combination of PRI and SIF increased prediction accuracy, and we identified the PRI_tot_+SIF_tot_-GPP model as the most applicable GPP estimation model.

**Table 2.** SIF_hs_/SIF_tot_ (W m^−2^ μm^−1^ Sr^−1^) and PRI_hs_/PRI_tot_ estimation models

Estimation model	Estimation formula	*R* ^2^	RMSE	*F*
PRI_hs_-GPP	GPP = −213.17 PRI_hs_+11.37	0.26**	8.5	98.05
PRI_tot_-GPP	GPP = −327.62 PRI_tot_+11.37	0.59**	6.3	415
SIF_hs_-GPP	GPP = 151.16 SIF_hs_+5.06	0.66**	5.8	541.5
SIF_tot_-GPP	GPP = 153.19 SIF_tot_−1.28	0.81**	4.4	1,173
PRI_hs_+SIF_hs_-GPP	GPP = 135.16 SIF_hs_-87.3 PRI_hs_+2.17	0.69**	5.5	317.4
PRI_tot_+SIF_tot_-GPP	GPP = 118.06 SIF_tot_-130.47 PRI_tot_−5.29	0.86**	3.7	847

*P < 0.05, **P < 0.01.

## Discussion

### GPP estimation model developed by PRI and SIF

As shown in Table 2, the PRI improved the *R*^2^ and RMSE between the SIF and GPP estimation models, both at hotspots and throughout the canopy, making the estimation model more efficient. A necessary condition for this improvement is that PRI is correlated with NPQ, an energy pathway that affects both photochemical and fluorescence quantum yields and regulates the lutein cycle. Many studies have confirmed a good relationship between the NPQ and PRI, with their linear *R*^2^ ranging from 0.6 to 0.9 [8,10–13,49–55], suggesting that the PRI is a good proxy for remote sensing of the NPQ. This study used more detailed spectral data from the rice canopy to justify the use of PRI to improve the spatial correlation of SIF-GPP at each half-hourly time step and to demonstrate the high degree of agreement between PRI and NPQ. These results were similar to those reported by Wang et al. [14]. In contrast, the present study is more detailed on a timescale and is applicable to crop canopies, where the daily variation in photosynthesis is more complex. The PRI+SIF-GPP estimation model used in this study can provide a reference for satellite remote sensing to track the carbon cycle over a large area. However, the shorter fertility of rice and other food crops was constrained by the observation timespan. Additional hyperspectral observations in the experimental field should be considered in the future to calibrate the estimation model.

### Comparison of hotspot and total canopy PRI/SIF

The results in Figs. 4 and 5 demonstrate the applicability of the two-leaf model to the rice canopy because of the favorable correlation between GPP and LAI. However, the effectiveness of the two-leaf model is heavily dependent on LAI [56,57]. We noticed that the largest GPP value occurred at approximately 12:00 in the half-hourly variation (Fig. 7). Whereas GPP presented a noon photosynthetic “inhibition” at 12:00, this phenomenon mirrored in both PRI and SIF, with the SIF_hs_ and PRI_hs_ being apparent. However, the “inhibition” decreased after distinguishing between sunlit and shaded canopy leaves because the solar radiation received by the other lower leaves tends to remain relatively low compared to the top leaves. Therefore, SIF_tot_ and PRI_tot_ are less likely to exhibit extremes around 12:00 [58].

The criterion for canopy normalization using the BRDF model is that it should be unaffected by the observed background reflections, which is supported by the results in Table 1. The reduction in the LAI influenced the calibration of the BRDF model for multi-angle rice observations. Under different LAI conditions, the correlations of PRIc and SIFc with GPP estimated using the two-leaf model were better than those of PRIh and SIFh. However, the background parameters were also part of the two-leaf model, according to Eq. 8. Badgley et al. [59] and Zeng et al. [60] recommended multiplying the normalized difference vegetation index (NDVI) by the near-infrared (NIR) reflectance to mitigate the detrimental impact of background reflections. Nevertheless, according to Lu et al. [61,62], the application of NDVI compensation diminishes the capacity of SIF to estimate GPP in broadleaf forests. In addition, the NDVI obtained from the observed spectra was not affected by the elimination of background information [58].

In this study, we used the PROSPECT model to calculate leaf reflectance instead of the maximum value from the multi-angle observation reflectance [26], which improved the accuracy of obtaining leaf reflectance based on the rice canopy. This is because leaf reflectance under direct sunlight is likely to not coincide with the limited observation angles [57]. Based on the establishment of the BRDF model in this study, optimization of the establishment of the BRDF model and hotspot selection will be considered, and the BRDF model will be fused into the two-leaf model to support leaf reflectance in the future. According to Figs. 6 and 8, SIF_tot_ has a better tracking performance than SIF_hs_, and PRI_tot_ can estimate GPP more accurately. The results of daily and half-hourly linear regression analyses show that SIF_tot_ and PRI_tot_ were more highly connected with GPP than SIFhs and PRI_hs_. Compared to SIF_hs_ and PRI_hs_, which primarily collected radiation signals from the parietal leaves, SIF_tot_ and PRI_tot_ captured the SIF and PRI from all rice canopy layers, whereas our vorticity flux observations were also targeted to the whole rice canopy; thus, SIF_tot_ and PRI_tot_ could show a stronger consistency with GPP. Table 2 and Fig. 10 indicate that SIF_tot_ and PRI_tot_ perform better at estimating GPP than SIF_hs_ and PRI_hs_; however, the scale conversion factor must be accounted for when constructing large-scale SIF+PRI-GPP models. To obtain SIF_tot_ and PRI_tot_ from existing satellite SIF and PRI data, several parameters, such as LAI, are required. However, these parameters are often coarse constants or 8-day products and require temporal interpolation to refine them. Experiments will be conducted in different agricultural regions to calibrate the satellite’s spectral observations for wider wavelength bands, gain a comprehensive understanding of the correlation between GPP variation and spectral reflectance in lutein and fluorescence-related absorption features, and provide a reference for parameter calibration of GPP models in global agricultural regions.

### Response of PRI and SIF to environmental factors

Environmental factors are complex for the PRI and SIF estimations of GPP; for example, *T* and VPD strongly affect the relationship between PRI and GPP. The main reason for this is that changes in temperature and humidity affect the activity of photosynthetic organs in leaves. In addition, this study found that both high and low *T* and VPD limited the PRI and SIF tracking of GPP (Table 1), suggesting that there is a threshold for the positive effects of both temperature and water vapor on remote sensing estimation studies of the rice canopy. The effect of temperature on vegetation index estimation in this study is similar to the results of some forest ecosystem studies [22,26], but there are differences in the effect of VPD [21,22], presumably due to differences in water sensitivity of leaf photosynthetic organs in different vegetation types. Subsequent studies on the threshold of water vapor effect on remote sensing estimation will be conducted for different crops. Different weather conditions lead to different PRI/SIF-GPP correlations; therefore, developing an empirical SIF+PRI-GPP model for crops throughout the fertility period remains a challenge.

## Conclusion

Our main objective was to investigate the SIF for estimating GPP when combined with PRI and to compare the PRI/SIF estimation capabilities at hotspots and the total canopy. The results of the validation dataset showed that the addition of PRI improved the accuracy and stability of SIF for estimating GPP, and the PRI/SIF of the total canopy performed better than that of the hotspot. These results demonstrate the feasibility of GPP estimation by combining SIF and PRI. Our study opens up a new perspective for accurately tracking crop photosynthetic processes using noninvasive sampling and provides a reference for studying the response of vegetation indexes to the environment.

## Data Availability

The data and model codes supporting the results of this study are available on reasonable request from the corresponding author (J.G.).
